# Chaos-Based Color Image Encryption with JPEG Compression: Balancing Security and Compression Efficiency

**DOI:** 10.3390/e27080838

**Published:** 2025-08-06

**Authors:** Wei Zhang, Xue Zheng, Meng Xing, Jingjing Yang, Hai Yu, Zhiliang Zhu

**Affiliations:** College of Software, Northeastern University, Shenyang 110167, China; xue0918login@163.com (X.Z.); xingmeng102@163.com (M.X.); 2310546@stu.neu.edu.cn (J.Y.); yuhai@mail.neu.edu.cn (H.Y.); zzl@mail.neu.edu.cn (Z.Z.)

**Keywords:** JPEG compression, image encryption, RSV pair recombination, evaluation indexes

## Abstract

In recent years, most proposed digital image encryption algorithms have primarily focused on encrypting raw pixel data, often neglecting the integration with image compression techniques. Image compression algorithms, such as JPEG, are widely utilized in internet applications, highlighting the need for encryption methods that are compatible with compression processes. This study introduces an innovative color image encryption algorithm integrated with JPEG compression, designed to enhance the security of images susceptible to attacks or tampering during prolonged transmission. The research addresses critical challenges in achieving an optimal balance between encryption security and compression efficiency. The proposed encryption algorithm is structured around three key compression phases: Discrete Cosine Transform (DCT), quantization, and entropy coding. At each stage, the algorithm incorporates advanced techniques such as block segmentation, block replacement, DC coefficient confusion, non-zero AC coefficient transformation, and RSV (Run/Size and Value) pair recombination. Extensive simulations and security analyses demonstrate that the proposed algorithm exhibits strong robustness against noise interference and data loss, effectively meeting stringent security performance requirements.

## 1. Introduction

In the era of highly computerized communication, information security has emerged as a critical area of research and public concern. While technological advancements have significantly enhanced convenience, they have also heightened the risks of personal information leakage. To mitigate the unauthorized access and exploitation of digital images, which often contain sensitive and private data, image encryption has garnered substantial attention as a robust protective measure [1]. Furthermore, the substantial storage requirements of image data during transmission necessitate efficient compression techniques to optimize space utilization and reduce transmission latency. Among the various compression algorithms available [2,3], the JPEG standard stands out for its exceptional performance. Consequently, to simultaneously address the dual challenges of image security and transmission efficiency, this study proposes an integrated approach that combines JPEG compression with advanced encryption techniques, thereby enhancing the overall security of digital images.

As an encryption method based on nonlinear dynamical systems, chaotic encryption technology has garnered significant attention in the field of information security in recent years. Chaotic systems exhibit properties such as sensitivity to initial conditions, pseudo-randomness, and ergodicity, which align closely with the requirements of encryption algorithms for randomness and unpredictability. These characteristics make chaotic systems an ideal tool for designing efficient encryption schemes. The chaos-based image encryption algorithm was first proposed by Fridrich in 1997 [4]. Since then, numerous related papers have been proposed [5,6,7,8,9,10,11,12,13,14]. Most of them encrypted the raw pixels of the image without considering the compression, which led to the larger data volume during transmission.

The JPEG international standard [15], established in 1993, has become a cornerstone technology widely adopted across various industries. In recent years, significant research efforts have been directed toward integrating JPEG compression with encryption algorithms [16,17,18,19,20]. However, encryption and compression are inherently contradictory. Compression algorithms leverage the high correlation within the image, while encryption algorithms aim to eliminate such correlations within the image data to achieve high security. Therefore, such algorithms often struggle to balance both security and compression efficiency. Based on the sequence of applying encryption and compression techniques, the combined processes can be systematically categorized into three distinct approaches: encryption before compression, encryption during compression, and encryption after compression. This classification provides a comprehensive framework for understanding and optimizing the interplay between data security and compression efficiency in digital image processing.

(1) Encryption before compression. Cryptographic schemes based on chaotic systems, deoxyribonucleic acid (DNA) encoding, or permutation mechanisms can be classified solely within this category [21,22,23,24,25,26,27,28]. Nevertheless, such approaches exhibit limited applicability in lossy compression frameworks, primarily stemming from two technical constraints. First, the initial encryption stage substantially diminishes inter-pixel spatial correlations within digital images, thereby reducing the inherent redundancy required for efficient compression. Second, the non-reversible nature of lossy compression introduces irreversible quantization errors during decoding, preventing precise reconstruction of original pixel values from cipher images.

(2) Joint encryption with compression. Encryption within compression frameworks represents an integrated approach combining cryptographic operations with compression processes [18,29,30,31,32,33,34,35,36,37]. This methodology enables encryption implementation across multiple stages of JPEG compression, including Discrete Cosine Transform (DCT), quantization, and entropy coding phases. Among existing solutions, this architecture has attracted substantial research attention due to its demonstrated performance advantages. Previous studies have proposed various implementation strategies. Li et al. [31] developed an 8 × 8 block transformation mechanism that integrates compression and encryption through alternating orthogonal transformations. While achieving 12.5% higher coding efficiency compared to conventional methods, this approach exhibits vulnerability to statistical analysis attacks due to its fixed block size constraint. He et al. [34] systematically evaluated three JPEG encryption schemes employing identical cryptographic algorithms but differing in integration points within the compression pipeline. Their comparative analysis revealed a 15–20% reduction in compression efficiency despite maintaining computational efficiency. More recently, Liang et al. [37] proposed a novel encryption framework featuring JPEG compatibility through entropy coding segment modification using cryptographic keys. However, their implementation demonstrates an inherent trade-off between security enhancement and file size expansion, with experimental results indicating a 25% average file size increase for optimal security configurations. Additionally, He et al. [38] combine the reversible data hiding scheme with an encrypted algorithm to ensure the security of JPEG images, and data extraction and information recovery can be performed independently.

(3) Post-compression encryption represents a sequential processing architecture where compression precedes cryptographic operations [39,40,41,42]. This methodology specifically targets the encryption of entropy-encoded bitstreams, maintaining compression compatibility through isomorphic codeword mapping that preserves original code lengths. While this approach demonstrates superior compression efficiency, it introduces potential format compliance issues. Specifically, the decoding process may generate blocks containing more than 64 elements, violating standard format specifications and potentially compromising system interoperability.

All of the aforementioned methods share a common issue: the trade-off between compression ratio and security. Specifically, it is difficult to simultaneously achieve high compression ratios and robust security. To address this challenge, we conducted an in-depth investigation into the various stages and different syntactic elements of the JPEG compression standard. Our study also examined the impact of encrypting these syntactic elements on both the final compression ratio and the security of the system.

This paper presents a novel integrated framework for simultaneous data compression and encryption, employing three encryption steps across three critical stages of image compression: Discrete Cosine Transform (DCT) transformation, quantization, and entropy encoding. The proposed scheme can be summarized as follows:

1. A 16 × 16 DCT transformation is initially executed during the DCT transformation phase, followed by applying a block segmentation algorithm to generate 8 × 8 sub-blocks.

2. The diffusion mechanisms are implemented through a dual approach in the quantization stage, including DC coefficient permutation and non-zero AC coefficient transformation algorithms.

3. An innovative RSV (Run/Size/Value) pair recombination algorithm is designed in the entropy encoding phase to significantly reduce spatial correlation among adjacent pixels.

4. Comparative analysis with existing methodologies demonstrates that the proposed framework achieves superior optimization between cryptographic robustness and compression efficiency, offering enhanced performance in both security and data compression domains.

The remainder of this paper is organized as follows. Section 2 provides a comprehensive overview of the JPEG compression standard and the foundational principles of chaotic systems. Section 3 elaborates on the proposed joint compression and encryption scheme, detailing its algorithmic framework and implementation specifics. Section 4 presents a systematic evaluation of the simulation experiments, including performance metrics and comparative analyses. Finally, Section 5 concludes the paper by summarizing the key findings, discussing their implications, and outlining potential directions for future research.

## 2. Preliminaries

### 2.1. JPEG Compression Standard

**Figure 1:**
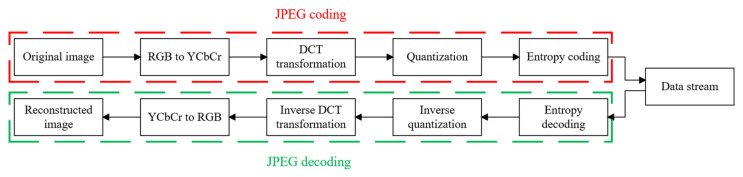
Basic process of JPEG compression and decompression.

The JPEG (Joint Photographic Experts Group) standard, established by the International Standards Organization (ISO) and the International Telegraph and Telephone Consultative Committee (CCITT) [43], represents the first international digital image compression standard for static images. As a widely adopted image compression standard, JPEG supports lossy compression, achieving significantly higher compression ratios compared to traditional compression algorithms. In practical implementations, the JPEG image encoding process employs a combination of Discrete Cosine Transform (DCT), Huffman encoding, and run-length encoding. For color images, the general architecture of the JPEG compression standard, as illustrated in Figure 1, consists of four primary stages: color space conversion, DCT transformation, quantization, and entropy coding. These stages collectively enable efficient compression while maintaining acceptable image quality.

In the execution process of the JPEG compression algorithm, different stages generate distinct syntactic elements. Encrypting these syntactic elements has varying impacts on data security and compression efficiency. The main four compression steps are as follows:

Color Space Conversion: While the JPEG standard is capable of compressing RGB components, it demonstrates superior performance in the luminance/chrominance (YUV) color space. For color images, an initial step involves the transformation of the RGB color space into the YUV color space. It is important to note that this color space conversion process is lossless, as it solely entails a mathematical mapping of pixel values from one color representation to another without any degradation in image quality.

Discrete Cosine Transform (DCT): DCT is a widely utilized coding technique in rate-distortion optimization for image compression. It serves as a mathematical transformation that converts spatial domain image representations into their frequency domain counterparts. This process can be conceptualized as a mapping operation that transforms an array of pixel values into a new array of frequency coefficients, effectively converting spatial light intensity data into frequency-based information. The transformation is applied to each image block independently, resulting in the concentration of energy into a limited number of coefficients, predominantly located in the upper-left quadrant of the transformed matrix. This energy compaction property is a fundamental characteristic of DCT, enabling efficient compression by prioritizing significant frequency components.

Quantization: The quantization process involves dividing each DCT coefficient by its corresponding value in a predefined quantization table, yielding the “quantized DCT coefficients.” During this stage, frequency coefficients are transformed from floating-point representations into integers, which simplifies subsequent encoding operations. It is evident that quantization introduces a loss of precision, as the data are reduced to integer approximations, thereby discarding certain information. In the JPEG algorithm, distinct quantization tables are applied to luminance (brightness) and chrominance components, reflecting their differing accuracy requirements. The design and selection of the quantization table play a critical role in determining the overall compression ratio, making it a pivotal factor in balancing image quality and compression efficiency.

Entropy coding: For the DC coefficient, Differential Pulse Code Modulation (DPCM) is employed. Specifically, the difference between each DC value in the same image component and the preceding DC value is computed and encoded. For the AC coefficients, Run Length Coding (RLC) is utilized to further reduce data transmission by encoding sequences of zero-valued coefficients. Subsequently, Huffman coding is applied to assign shorter binary codes to symbols with higher probabilities of occurrence and longer binary codes to symbols with lower probabilities, thereby minimizing the average code length. Different Huffman coding tables are utilized for the DC and AC coefficients. Additionally, distinct Huffman coding tables are employed for luminance and chrominance components. Consequently, four Huffman coding tables are required to complete the entropy coding process.

Decompression is the inverse process of JPEG compression encoding. The primary steps involved in JPEG decompression are illustrated in Figure 1.

### 2.2. Chaotic System

A chaotic system is defined as a deterministic system that exhibits seemingly random and irregular motion, characterized by behaviors that are uncertain, non-repeatable, and unpredictable. This phenomenon, known as chaos, has garnered significant attention and is widely applied in the field of image encryption due to its inherent complexity and sensitivity to initial conditions. Among the foundational discoveries in chaos theory, the Lorenz system stands as the first identified chaotic attractor, marking a pivotal milestone in the study of chaotic dynamics [44]. This system represents the earliest dissipative system demonstrating chaotic motion, as revealed through numerical experiments. It is a three-dimensional system governed by three parameters, which collectively contribute to its rich and intricate dynamical behavior.

Low-dimensional chaotic systems are characterized by the presence of only one positive Lyapunov exponent, which results in a relatively limited key space. This limitation makes them vulnerable to contemporary brute-force attacks. To address this issue, this paper employs the four-dimensional hyperchaotic Lorenz system proposed in Ref. [45]. This hyperchaotic system features two positive Lyapunov exponents, thereby enhancing the complexity and robustness of the chaotic behavior. The system’s definition is as follows:

(1) $$\left\{\begin{array}{l} \frac{\partial x}{\partial t}=a(y-x)+w,\\ \frac{\partial y}{\partial t}=cx-y-2xz,\\ \frac{\partial z}{\partial t}=2x^{2}-bz,\\ \frac{\partial w}{\partial t}=yz-dw. \end{array}\right.$$

where a, b, c, d are system parameters. When a = 10, b = 8/3, c = 28, and d = 2, the Lyapunov exponents of Equation (1) are $\lambda_{1}\;=\;2.0438$, $\lambda_{2}\;=\;1.9735$, $\lambda_{3}\;=\;-2.1918$, and $\lambda_{4}\;=\;-35.4927$; two of these Lyapunov exponents are positive. Under these conditions, Equation (1) exhibits hyperchaotic motion.

### 2.3. Analysis of Syntactic Elements for Compression and Encryption

**Figure 2:**
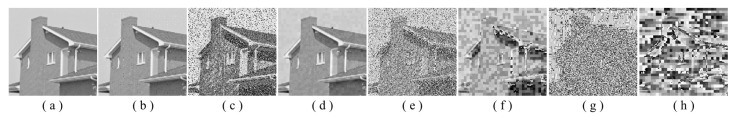
The visualized results of encrypting different syntax elements in distinct steps. (**a**) Original image. (**b**) The first plain value (FPV) in each block with size 8 × 8 is encrypted. (**c**) The rest of the plain value excluding the first (RPV) in each block with size 8 × 8 is encrypted. (**d**) The DC coefficient after the DCT transformation (DCADCT) is encrypted. (**e**) The AC coefficient after the DCT transformation (ACADCT) is encrypted. (**f**) The DC coefficient after the quantification (DCAQ) is encrypted. (**g**) The AC coefficient after the quantification (ACAQ) is encrypted. (**h**) The DC coefficient after DPCM coding (DCADPCM) is encrypted.

Two syntax elements have been universally accepted in the JPEG compression process: DC coefficients and AC coefficients. The DC and non-zero AC coefficients are encrypted in distinct phases, each significantly influencing communication efficiency, bit expansion, and perceptual security to varying degrees. By combining the BLAKE-256 hash function with the Lorenz hyperchaotic system, test keys are generated, exhibiting high stochasticity within the range [0, 31]. Extensive empirical studies, conducted using a simple XOR (Exclusive OR) operation, confirm the fairness of the process. The visual results are shown in Figure 2, with a detailed analysis provided in Table 1.

As shown in Figure 2, the detailed information of the original image is concealed within a ciphertext image, where only part of the plain pixel values are encrypted. All information is encoded in the encrypted image, resulting in block effects, with the DC coefficients being encrypted after the DCT transformation. In contrast, the AC coefficients are encrypted following the DCT transformation, which enhances data security while still allowing for recognition of object contours. It is noteworthy that certain syntax elements, such as DC and AC coefficients after quantization and DC coefficients after DPCM coding, are selectively chosen for encryption, further strengthening information privacy.

## 3. Methodology

**Figure 3:**
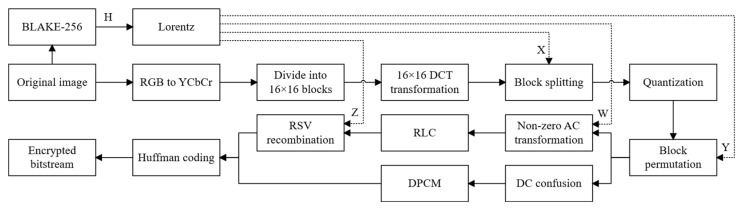
Encryption framework of the proposed scheme.

In the process of simultaneously performing image compression and encryption, the compression operation and the encryption operation can interfere with each other. The reason lies in the fact that the goal of encryption is to eliminate the correlation between image pixels, while compression exploits these correlations to reduce the image size. A poorly designed encryption algorithm can significantly degrade the compression ratio. Through a comprehensive analysis of the effects of different syntactic elements in the image compression process on both encryption and compression performance, we propose a joint compression and encryption algorithm based on chaos theory. This algorithm consists of the following four steps, as shown in Figure 3.

### 3.1. Key Scheming

Secret keys play a crucial role in cryptographic systems, serving as the cornerstone of their security. According to the widely accepted Kerckhoffs’s principle, the robustness of a cryptosystem should remain intact even if all aspects of the system, excluding the secret key, are publicly disclosed. This principle underscores the importance of key secrecy in ensuring the overall security of encryption mechanisms. In the proposed scheme, the four initial values of the Lorenz hyperchaotic system are designated as the cryptographic key. Prior to iterating the chaotic system to generate pseudo-random sequences, the BLAKE-256 hash function is utilized to compute a 256-bit hash value, denoted as *H*. This hash value is subsequently employed to modify the four initial values of the Lorenz hyperchaotic system, resulting in the generation of four updated initial values. These modified values are then substituted into the Lorenz hyperchaotic system for iterative computation, producing four pseudo-random key sequences, X, Y, Z, and W. This approach enhances the security and randomness of the key generation process. The specific process is as follows:

**Step 1:** The 256-bit hash value *H* is partitioned into 32 segments, each comprising 8 bits, expressed as $H\;=\;h_{1}h_{2}\ldots h_{32}$, where each hi falls within the range [0, 255]. Subsequently, the initial values of the Lorenz hyperchaotic system, denoted as $x_{0}$, $y_{0},\;z_{0},$ and $w_{0}$, are modified using Equation (2). This step ensures the initialization of the chaotic system with updated values derived from the hash segments.

(2) $$\left\{\begin{array}{l} x_{0}^{\prime }=x_{0}+\frac{\left(h_{1}⊕h_{2}⊕\ldots ⊕h_{8}\right)}{256},\\ y_{0}^{\prime }=y_{0}+\frac{\left(h_{9}⊕h_{10}⊕\ldots ⊕h_{16}\right)}{256},\\ z_{0}^{\prime }=z_{0}+\frac{\left(h_{17}⊕h_{18}⊕\ldots ⊕h_{24}\right)}{256},\\ w_{0}^{\prime }=w_{0}+\frac{\left(h_{25}⊕h_{26}⊕\ldots ⊕h_{32}\right)}{256}. \end{array}\right.$$

**Step 2:** The Lorenz hyperchaotic system is iterated 10,000 times using the updated initial values *x*_0_′, *y*_0_′, *z*_0_′, and *w*_0_′. Each iteration can obtain four values *x_i_*, *y_i_*, *z_i_*, and *w_i_*, *i* = 1, 2, …, 10,000. Let *M* and *N* represent the height and width of the image, respectively. These generated values are subsequently preprocessed using Equation (3) to ensure their suitability for further cryptographic operations.

(3) $$\left\{\begin{array}{l} x_{i+1}=((x_{i}-\left\lfloor x_{i}\right\rfloor )\times 10^{14})\mathrm{mod}(M\times N),\\ y_{i+1}=((y_{i}-\left\lfloor y_{i}\right\rfloor )\times 10^{14})\mathrm{mod}(M\times N),\\ z_{i+1}=((z_{i}-\left\lfloor z_{i}\right\rfloor )\times 10^{14})\mathrm{mod}(M\times N),\\ W=((w_{i}-floor(w_{i})\times 10^{14})\mathrm{mod}(M\times N). \end{array}\right.$$

**Step 3:** All values within the pseudo-random sequences *X*, *Y*, *Z*, and *W* are converted into binary format to govern the subsequent encryption process. Consequently, the encryption key comprises two distinct components: (1) a 256-bit hash value *H*, and (2) the four initial values of the Lorenz hyperchaotic system, denoted as *X*_0_, *Y*_0_, *Z*_0_, and *W*_0_. This dual-component structure ensures a robust and secure foundation for the encryption mechanism.

### 3.2. DCT Transformation Stage Encryption

The first-stage encryption process is implemented following the Discrete Cosine Transform (DCT), which begins by partitioning the image into 16 × 16 blocks and subsequently performing the DCT transformation on these blocks. However, since the standard quantization table specified by the JPEG compression standard is designed for 8 × 8 blocks, each 16 × 16 block (denoted as *B*16) is further divided into four 8 × 8 blocks (denoted as *B*8) after the DCT transformation. As a result, the 256 coefficients of each 16 × 16 block are distributed across four 8 × 8 blocks. This division ensures compatibility with the standard JPEG encoding process, which can be seamlessly applied in subsequent steps. Below is the fundamental procedure of the block-splitting algorithm:

**Step 1:** The 16 × 16 block (*B*16) is transformed into a one-dimensional sequence using zigzag scanning. This sequence is then evenly divided into two parts, denoted as *B*16_1 and *B*16_2, each containing 128 coefficients.

**Step 2:** The coefficients in *B*16_1 are allocated to four 8 × 8 blocks. For each allocation, two bits from the pseudo-random key stream *X* are used to determine the target 8 × 8 block, while three additional bits from *X* specify the number of coefficients to be assigned to the selected block. After each allocation, the corresponding five bits in *X* are removed. This process is repeated until all coefficients in *B*16_1 are distributed, ensuring that each 8 × 8 block contains 32 coefficients.

**Step 3:** The coefficients in *B*16_2 are allocated to the four 8 × 8 blocks using the same procedure as described in Step 2. Upon completion, each 8 × 8 block contains a total of 64 coefficients.

**Step 4:** Each one-dimensional 8 × 8 block (*B*8) is transformed back into a two-dimensional 8 × 8 block through the inverse zigzag scanning process.

During the block-splitting process, the number of 8 × 8 blocks to which the DC coefficient (the first coefficient of the original *B*16) is assigned is recorded. This information is utilized in the encryption algorithm during the quantization stage. The above block-splitting algorithm is applied to each 16 × 16 block, thereby completing the encryption algorithm in the DCT transformation stage.

### 3.3. Quantization Stage Encryption

In the second encryption stage, block permutation is performed following the quantization process to achieve encryption scrambling and diffusion effects. Specifically, the DC coefficients undergo confusion, while the signs of the non-zero AC coefficients are transformed. These operations collectively enhance the security and robustness of the encryption scheme by introducing additional layers of complexity and unpredictability.

#### 3.3.1. Blocks Permutation

To further enhance the chaotic nature of the encrypted image, a block permutation operation is introduced after the quantization process. This operation disrupts the original order of the 8 × 8 blocks based on the pseudo-random key sequence *Y* before advancing to the entropy encoding stage. The permutation algorithm employed is a random permutation method. In the context of our encryption scheme, *B* represents the original sequence of all 8 × 8 blocks, *n* denotes the total number of 8 × 8 blocks, and the random integers required for permutation are derived from the pseudo-random key sequence *Y*. The algorithm is described in Algorithm 1.
**Algorithm 1** Blocks permutation algorithm**Input:** All 8 × 8 blocks after quantization; *Y*.**Output:** The result of block permutation.1:$r\;=\;\left\lfloor \log_{2}n\right\rfloor$;2:**where** i ≤n **do**3: *j*
← pick r bits from *Y*, and convert to decimal;4: exchange B[i] and B[j];5: remove the first r bits from Y.6:**end while**

#### 3.3.2. DC Coefficients Confusion

After the permutation of blocks, the index sequence of the DC coefficients within the original 16 × 16 blocks undergoes a transformation. Consequently, the index sequence of the 8 × 8 blocks containing the DC coefficients from the original 16 × 16 blocks must be updated in accordance with the permutation vector S0. To further enhance the diffusion and obfuscation characteristics of the encryption scheme, an XOR calculation is applied to the DC coefficients, following the order they appear after the block permutation. The calculation is formulated as follows:

(4) $$\left\{\begin{array}{l} dc_{\left(1\right)}\;=\;dc_{\left(1\right)}\;\;\;\;\;\;\;\;\;\;\;\;\;\;\;\;\;\;\;\;\;\;\;\;\;\;\;\;\;\;\;\;\;\;\;\;\;\;\;\;\;\;\;\;\\ dc_{\left(i\right)}\;=\;dc_{\left(i\right)}⊕dc_{\left(i-1\right)}⊕\ldots ⊕dc_{\left(1\right)},\; \end{array}\right.$$

where $dc_{\left(i\right)}$ represents the DC coefficient of i-th 8 × 8 block,$\;i\;=\;2,\;3,\ldots ,\;\left(\left\lceil \frac{M-1}{16}\right\rceil +1\right)\times ([(N-1)/16]+1)$. In the decryption process, the original value of the DC coefficient can be restored by Equation (5).

(5) $$\left\{\begin{array}{l} dc_{\left(1\right)}=dc_{\left(1\right)}\\ dc_{\left(j\right)}=dc_{\left(j\right)}⊕dc_{\left(j-1\right)}⊕\ldots ⊕dc_{\left(1\right)}, \end{array}\right.$$

where $j=2,3,\ldots ,\left(\left\lceil \frac{M-1}{16}\right\rceil +1\right)\times ([(N-1)/16]+1)$.

#### 3.3.3. Non-Zero AC Coefficients Sign Transformation

After quantization, the non-zero AC coefficients are identified and extracted. The encryption effect is achieved by altering the order of these non-zero AC coefficients using the pseudo-random key stream W, as well as modifying their values through sign transformation. This step preserves the distribution of zero-valued AC coefficients, ensuring that the length of the encrypted bitstream remains largely unaffected. The transformation is computed as follows, as defined in Equation (6):

(6) $$ac_{k}\;=\;(-1)\times ac_{k}$$

where $ac_{k}$ represents the value of the k-th non-zero AC coefficient. Once the sign transformation of the non-zero AC coefficients is completed, these coefficients are restored to their original positions. This ensures that the structural integrity of the data is maintained while achieving the desired encryption effect.

### 3.4. Entropy Coding Stage Encryption

The primary objective of the encryption operation in the entropy encoding stage is to strengthen the correlation removal capability of the encryption scheme while preserving format consistency. Although the encryption algorithms in the first two stages contribute to security, they do not effectively eliminate the correlation among 8 × 8 blocks. To address this limitation, an RSV (Run/Size/Value) recombination strategy is implemented during the entropy coding stage. This strategy enhances the decorrelation of the data, further improving the security and robustness of the encryption scheme.

The primary objectives of the encryption at this stage are to maintain format consistency and to reduce the correlation within the image. Prior to entering the entropy encoding stage, zigzag scanning is applied to each 8 × 8 block to separate the DC and AC coefficients. For the DC coefficients, Difference Pulse Code Modulation (DPCM) is employed. For the AC coefficients, Run Length Coding (RLC) is utilized, converting them into data pairs known as RSV (Run/Size/Value) pairs. These RSV pairs are then subjected to Huffman encoding to generate variable-length codes. To enhance security, we propose encrypting these RSV pairs through an RSV pair recombination algorithm, and the specific realization is given in Algorithm 2. This recombination process is governed by the pseudo-random key sequence Z. The steps of the algorithm are outlined as follows:

**Step 1:** AC coefficients with a value of zero are referred to as zero-value blocks, meaning their RSV pairs consist solely of the end identifier (0, 0). Blocks containing non-zero AC coefficients are termed non-zero blocks, indicating the presence of non-zero RSV pairs. For non-zero blocks, all end identifiers are removed.

**Step 2:** All blocks are evenly divided into two parts, denoted as *B*_1_ and *B*_2_, with each block in *B*_1_ corresponding sequentially to a block in *B*_2_.

**Step 3:** A block from *B*_1_ and its corresponding block from *B*_2_ are sequentially processed, and the following conditions are evaluated:

***1.* ** If both blocks are zero-valued blocks, their values remain unchanged.

***2.* ** If one block is zero-valued and the other is non-zero-valued, the two blocks are swapped. Subsequently, all RSV pairs of the new non-zero block undergo cyclic shifting to disrupt their original distribution pattern. The shift amount is determined by the corresponding key sequence *Z*.

***3.* ** If both blocks are non-zero blocks, the number of RSV pairs in the first block is denoted as RSVnum. All RSV pairs from both blocks are concatenated and cyclically shifted based on the key sequence *Z*. The difference between the number of RSV pairs newly assigned to the first block and RSVnum is recorded as *T*. Initially, the division is performed according to the original number of RSV pairs in each block, with *T* = 0. Since the number of AC coefficients in each block cannot exceed 63 (to maintain format compatibility and avoid entropy decoding issues), the number of AC coefficients in the newly assigned blocks must be verified. If the number of AC coefficients in either block exceeds this limit, the division point is adjusted as follows:

(1) If neither block exceeds the limit, the division is performed at the original cutting point, and T=0.

(2) If the first block exceeds the limit, the division point is shifted left until both blocks are within the acceptable range. The shift amount is recorded as a negative value.

(3) If the second block exceeds the limit, the division point is shifted right until both blocks are within the acceptable range. The shift amount is recorded as a positive value.

**Figure 4:**
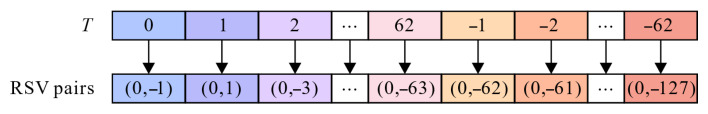
RSV pairs corresponding to different T values.

The value of T is converted into an RSV pair, and the corresponding relationship between the T value and the RSV pair is established using a variable-length integer coding table, as shown in Figure 4. The RSV pair is appended to the end of the first block. The condition for determining whether the value is out of range is that it should not exceed 62.

**Figure 5:**
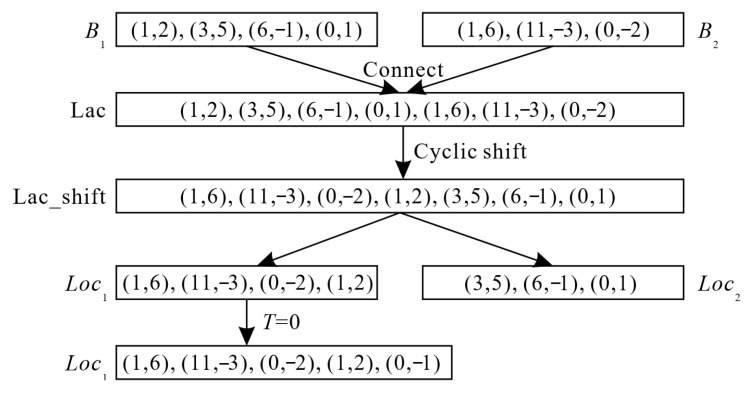
A simple example of Step 3.

To better illustrate the RSV pair recombination process, a simple example is provided in Figure 5. In this example, *B*_1_ and *B*_2_ are two non-zero AC blocks with the original end identifier removed. *Loc*_1_ and *Loc*_2_ represent the results of the RSV pair recombination.

**Step 4:** Repeat the steps outlined in Step 3 until all blocks in *B*_1_ and *B*_2_ are complete.

**Step 5:** Add end identifiers to all non-zero blocks after recombination. If the number of AC coefficients in the block reaches the upper limit, no additional end identifiers will be added.

**Step 6:** Rearrange the positions of all blocks in a circular manner, with the displacement controlled by the key sequence Z. This rearrangement only moves the RSV pairs within the block, while the position of the encoded DC coefficient remains unchanged. The RSV recombination strategy is shown in Algorithm 2.
**Algorithm 2** RSV recombination strategy**Input:** All RSV pairs; *Z*.**Output:** The result of RSV recombination.1:Remove the end identifier of each block except comprise merely [0, 0];2:The adjacent blocks are denoted *B*_1_ and *B*_2_;3:**if** zero value exhibits in *B*_1_ and *B*_2_ **then**4:   Remain unchanged;5:**else if** zero value exhibits *B*_1_ or *B*_2_ **then**6:   Swapping *B*_1_ and *B*_2_ and connecting them in Lac;7:   All RSV of Lac are shifted cyclically according to *Z*;8:**else**9:   Calculate the number of RSV in *B*_1_, denoted as RSVnum;10:   Connecting *B*_1_ and *B*_2_ in Lac;11:   All RSV of Lac are shifted cyclically according to *Z*;12:   *F_n_* ← The number of RSV pairs in the first block;13:   *S_n_* ← The number of RSV pairs in the second block;14:   times ← Number of moves;15:   **if** *F_n_* ≤63**then**16:    Split Lac using original point;17:    *T* ← The difference in the number of RSV pairs in the first block with RSVnum;18:    *T* ← 0;19:    The RSV pair corresponding to *T* is spliced into the rear of the first block;20:   **else if** *F_n_* >63 **then**21:    Split point is left relocated until *F_n_*  ≤ 63;22:    *T* ← times23:    The RSV pair corresponding to *T* is spliced into the rear of the first block;24:   **else if**
*S_n_*
>63 **then**25:    Split point is right relocated until *S_n_* ≤63;26:    *T* ← times27:    The RSV pair corresponding to *T* is spliced into the rear of the first block;28:Insert the end identifier in each block;29:All RS pairs in each block are shifted cyclically according to *Z*.

## 4. Simulation Results

**Figure 6:**
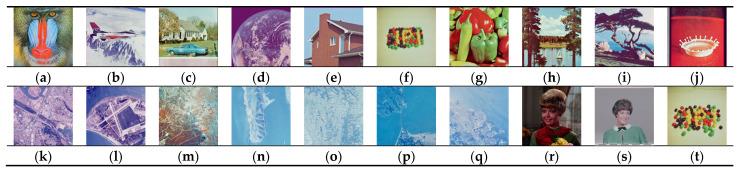
Test images. (**a**) Mandrill; (**b**) Airplane; (**c**) Car; (**d**) Earth; (**e**) House; (**f**) Jelly beans; (**g**) Peppers; (**h**) Sailboat; (**i**) Tree; (**j**) Splash; (**k**) San Diego; (**l**) San Diego2; (**m**) Woodland Hills, Ca.; (**n**) San Diego3; (**o**) San Diego4; (**p**) San Francisco; (**q**) Foster City, Ca.; (**r**) Female; (**s**) Female2; and (**t**) Jelly beans2.

In this section, we assess the encryption security and compression performance of the proposed encryption scheme. For the experimental setup, the parameters of the Lorenz hyperchaotic system are defined as $a\;=\;10,\;b\;=\frac{8}{3},\;c\;=\;28,\;and\;d\;=\;2$. The initial states of the system are set to *x*_0_ = 0, *y*_0_ = 11, *z*_0_ = 14, and *w*_0_ = 5. Experiments are conducted on the widely used image database: the USC-SIPI image database [46]. A total of twenty color images, as depicted in Figure 6, are selected as experimental samples. The experiments are performed in a Python 3.9 environment on a 64-bit operating system with an Intel Core i7-10700K processor running at 2.90 GHz and 16 GB of RAM. The performance of the proposed scheme is compared with Ref. [31] and two existing schemes referenced in [18], providing a comprehensive evaluation of its effectiveness.

### 4.1. Encryption Security

Image encryption algorithms necessitate a dual assurance of both perceptual security and cryptographic security. Perceptual security pertains to the degree of perceptual distortion introduced in the cipher image relative to the plain image. This distortion is quantitatively assessed using metrics such as the Peak Signal-to-Noise Ratio (PSNR) and Structural Similarity (SSIM). High values of PSNR and close-to-unity SSIM indicate lesser distortion, whereas lower PSNR values and significant deviations in SSIM from unity signify greater distortion, reflecting a stronger perceptual security in the encrypted image.

Furthermore, the mean square error (MSE) serves as an objective measure to compute the PSNR. Given a plain image I and its corresponding cipher image K, both of size M × N, the MSE and PSNR are defined as follows:

(7) $$MSE=\frac{1}{m\times n}\sum_{i=0}^{m-1}\sum_{j=0}^{n-1}[I(i,j)-K(i,j){]}^{2}.$$

(8) $$PSNR=10\times \log_{10}(\frac{MAX_{I}^{2}}{MSE}).$$

where *MAX*_I_ is the maximum possible pixel value of the image. If each pixel is represented by an 8-bit binary, the value of *MAX*_I_ is 255.

The Structural Similarity Index (SSIM) is a metric used to evaluate the similarity between two images by assessing three key aspects: luminance (brightness), contrast, and structure. Let *x* represent the plain image and *y* represent the cipher image. The SSIM is defined as follows:

(9) $$SSIM\left(x,y\right)\;=\;\frac{\left(2\times \mu_{x}\times \mu_{y}+c_{1})\times (\sigma_{xy}+c_{2}\right)}{\left(\mu_{x}^{2}\times \mu_{y}^{2}+c_{1})\times (\sigma_{x}^{2}+\sigma_{y}^{2}+c_{2}\right)}.$$

where $\mu_{x}$ and $\mu_{y}$ represent the average values of x and y, respectively. $\sigma_{x}$ and $\sigma_{y}$ denote the standard deviations of *x* and *y*, respectively. $\sigma_{xy}$ is the covariance of *x* and *y*. $c_{1}$ and $c_{2}$ are constants to avoid system errors with a denominator of 0.

**Figure 7:**
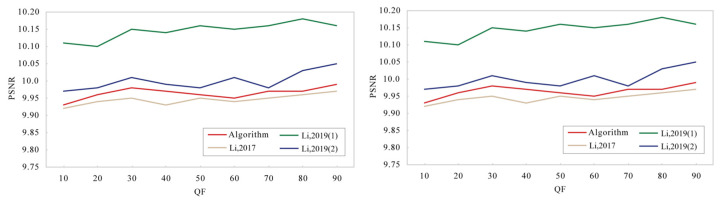
Perceptual security results (Proposed Algorithm, Ref. [18], and Ref. [31]).

In the proposed encryption methods, we encrypted twenty images using different quality factor (QF) values. The average PSNR and SSIM values for these encrypted images are presented in Figure 7. In contrast, the first scheme described in Reference [18] solely employs encryption during the transformation and quantization stages. As evident from Figure 7, our proposed method yields lower PSNR and SSIM values compared to the two schemes presented in Reference [18]. This indicates that our encryption method possesses superior image distortion capabilities.

#### 4.1.1. Brute-Force Attack

A brute-force attack involves attempting all possible key values in order to recover the actual key and successfully execute the attack. To ensure image security, it is essential that the key space of a cryptosystem be sufficiently large. In our encryption scheme, the encryption keys consist of a 256-bit random hash value, denoted as σ, generated by the BLAKE-256 algorithm, and four initial states (*x*_0_, *y*_0_, z_0_, and *w*_0_) of the Lorenz chaotic system. Given a precision of 10^14^ for the four initial values, the key space size is 10^56^. The total key space size, therefore, is 2^256^ × 10^56^, which is computationally infeasible for attackers to brute force. Additionally, for different plaintext images, BLAKE-256 produces distinct 256-bit random hash values, and the initial values of the Lorenz system vary, further increasing the difficulty of a potential attack.

#### 4.1.2. Key Sensitivity Analysis

Encryption key leads to significant variations in both the generated key sequence and the resultant cipher image during the encryption and decryption processes. This property ensures that minor alterations in the key yield substantial differences in the encrypted and decrypted outputs. Key sensitivity can be evaluated based on two criteria:

(1) A small variation in the encryption key, when applied to the same plaintext image, should produce a drastically different ciphertext image.

(2) A minimal alteration to the encryption key must generate a cipher image with statistically indistinguishable similarity to random noise, while any decryption attempt using the perturbed key should yield only non-recognizable, noise-corrupted content.

In the proposed scheme, the encryption keys consist of two components: (1) a 256-bit random hash value σ derived from the BLAKE-256 hash function, which is generated using both the plaintext image and a random value; and (2) the four initial states *x*_0_, *y*_0_, *z*_0_, and *w*_0_. During the key sensitivity analysis, the 256-bit hash value σ remains constant as there are no changes to the plaintext image. Therefore, to assess the impact of key sensitivity, we focus on modifying these four initial states, which allows us to observe the variations in the encryption and decryption results.

To evaluate the first case of key sensitivity, we introduce slight modifications to the four initial values *x*_0_ = 0, *y*_0_ = 11, *z*_0_ = 14, and *w*_0_ = 5 by changing each value in turn. Specifically, we generate four distinct keys as follows: key1 (*x*_0_ = 0.00000001, *y*_0_ = 11, *z*_0_ = 14, *w*_0_ = 5), key2 (*x*_0_ = 0, *y*_0_ = 11.00000001, *z*_0_ = 14, *w*_0_ = 5), key3 (*x*_0_ = 0, *y*_0_ = 11, *z*_0_ = 14.00000001, *w*_0_ = 5), and key4 (*x*_0_ = 0, *y*_0_ = 11, *z*_0_ = 14, *w*_0_ = 5.00000001). These keys are then used to encrypt various images in accordance with the proposed algorithm.

The correlation coefficients between the ciphertexts produced by the original key and each of the slightly altered keys are presented in Table 2. The observed low correlation coefficients demonstrate that even small changes in the initial key lead to significant variations in the encrypted images, thereby confirming that the proposed scheme satisfies the first condition of key sensitivity.

**Figure 8:**
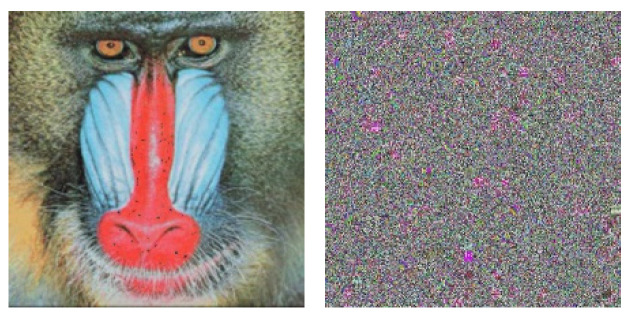
Key sensitivity analysis for the decryption process: (**left**) decrypted image via the correct key, (**right**) decrypted image via the modified key.

For the second case of key sensitivity, we perform decryption using both the correct key and a slightly altered key on the ciphered Mandrill image. The decrypted results are shown in Figure 8. As expected, the decryption using the slightly modified key does not produce the correct image, highlighting the sensitivity of the scheme to key changes. This demonstrates that even a minor variation in the key prevents the recovery of the original image, thereby satisfying the second condition of key sensitivity.

#### 4.1.3. Differential Attack

Differential attack is a type of chosen-plaintext attack and serves as a crucial method for analyzing the plaintext sensitivity of encryption algorithms. In this type of attack, the adversary aims to identify statistical relationships by calculating the differences between a plaintext image and its corresponding ciphertext [47]. If the algorithm is vulnerable, small changes in the plaintext should result in noticeable patterns or predictable differences in the encrypted output.

The Number of Pixel Change Ratio (NPCR) and Unified Average Change in Intensity (UACI) are two important statistical parameters widely used to evaluate the robustness of image encryption schemes against differential attacks. NPCR measures the ratio of differing pixel values between two ciphertexts generated from the original plaintext image and the altered plaintext image, where a single pixel value is modified. Specifically, it quantifies the proportion of pixels in the ciphertext that have changed when the plaintext is slightly modified. A higher NPCR value indicates a greater degree of change between the ciphertexts, signifying a more secure encryption scheme that resists differential attacks effectively. UACI calculates the average change in intensity between the pixels of two ciphertexts derived from the original and the slightly altered plaintext images. This metric measures the average change density across the entire encrypted image. For higher security, the value of UACI should be close to 33%, which represents a balanced and significant change in the ciphertext corresponding to slight changes in the plaintext. The calculations of these two parameters are defined as follows:

(10) $$D\left(i,j\right)\;=\;\left\{\begin{array}{l} 0,if\;C^{1}(i,j)\;=\;C^{2}(i,j)\\ 1,if\;C^{1}(i,j)\neq C^{2}(i,j) \end{array}\right..$$

(11) $$NPCR:N\left(C^{1},C^{2}\right)=\frac{\sum_{i=1}^{M}\sum_{j=1}^{N}D(i,j)}{M\times N}\times 100\%.$$

(12) $$UACI:U\left(C^{1},C^{2}\right)=\frac{\sum_{i=1}^{M}\sum_{j=1}^{N}\frac{\left|C^{1}(i,j)-C^{2}(i,j))\right|}{255}}{M\times N}\times 100\%.$$

where $C^{1}$ and $C^{2}$ are two cipher images corresponding to two plain images differing by one single pixel. The range of i is 1≤i≤M and the range of j is 1≤j≤N.

The mean NPCR and UACI results for our encryption algorithm are listed in Table 3. For comparison, the NPCR and UACI values for the cipher images generated by the two encryption schemes described in Ref. [18] and Ref. [31] are also included in Table 4. The encryption scheme in Ref. [18] uses key generation machines that rely on ordinary images. From the analysis of experimental results, all encryption schemes have defense capabilities against differential attacks.

#### 4.1.4. Statistical Attack

Correlation analysis involves examining the relationship between two or more variables to assess their degree of correlation. In the context of images, adjacent pixels typically exhibit high correlation, meaning that a pixel often contains information about its neighboring pixels. Attackers can exploit this by analyzing specific data segments between plaintext and ciphertext images, attempting to predict the plaintext without knowledge of the encryption key. As a result, image encryption schemes must effectively reduce the correlation between image pixels. In Table 5 and Table 6, we measure correlation in three directions: horizontal, vertical, and diagonal. The correlation coefficients for various images are provided, with the Quality Factor (QF) set to 50.

In Table 6, the correlation between adjacent pixels in the image encrypted using our algorithm is significantly lower than other methods. This reduction is attributed to the added entropy encoding stage in our encryption process. When compared to the three schemes, our algorithm effectively diminishes the correlation between adjacent pixels, particularly in the vertical direction. Table 7 presents the correlation values for the original ‘Mandrill’ image and the ciphertexts produced by various encryption schemes.

#### 4.1.5. Information Entropy Analysis

Information entropy is an important metric used to quantify the randomness of the pixels in the images [48]. Information entropy is close to eight after encrypted gray-image mapping the effectiveness of the proposed encryption algorithm. The results of information entropies with different algorithms in distinct images are exhibited in Table 8, in which the information entropy of our presented encryption scheme is higher than Ref. [31]. Moreover, the result of the proposed algorithm is close to, and slightly higher than, Ref. [18], which has proven impressive for our algorithm in terms of ciphertext attacks to some extent.

### 4.2. Compression Performance

**Figure 9:**
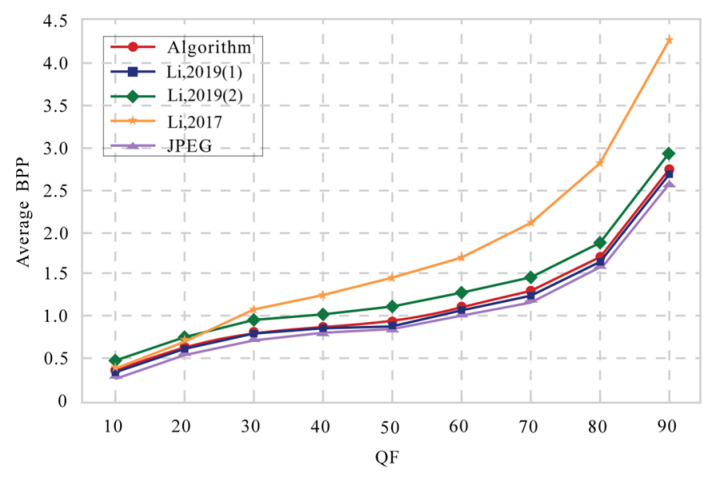
The QF-BPP curves with different encryption schemes: nine test images (Proposed Algorithm, Ref. [18], and Ref. [31]).

**Figure 10:**
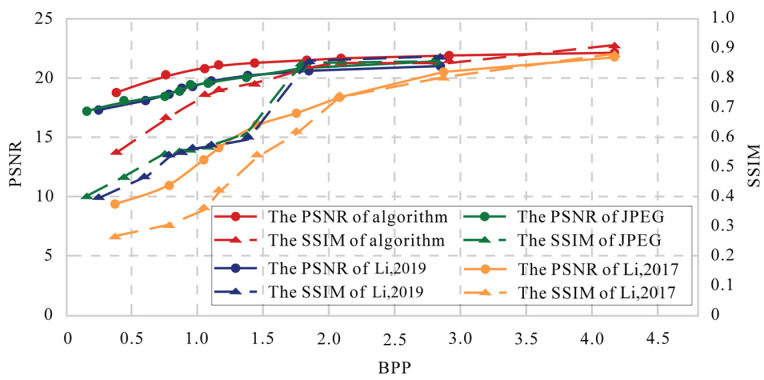
The PSNR-BPP-SSIM curves with different compression schemes(Proposed Algorithm, Ref. [18], and Ref. [31]).

Our encryption scheme is integrated into the lossy JPEG compression process, with compression performance influenced by the Quality Factor (QF) values. A higher QF value results in less compression, leading to a larger bitstream size. To evaluate compression performance, we use the bits per pixel (BPP) metric. The average BPP values are calculated for the reconstructed images. The experimental images used are the nine shown in Figure 6. In Figure 9, we present the QF-BPP curves for various encryption schemes, with QF values ranging from 10 to 90. From the average QF-BPP curves shown in Figure 9, the compression performance of our proposed scheme is better than Ref. [31] and the second scheme in Ref. [18], but worse than the first scheme in Ref. [18]. This can be attributed to the introduction of the RSV (Replaced and Reassembled Variable) in our approach, which increases the bitstream size. Unlike the first scheme in Ref. [18], our encryption does not occur during the entropy encoding stage. In contrast, the second scheme in Ref. [18] adds an additional RSV pair for each block during the entropy encoding encryption process, along with an EOB (End-of-Bit) embedding step, resulting in a larger bitstream size. However, our scheme only incorporates half of the additional RSV pairs, leading to better compression performance compared to the second scheme in Ref. [18]. Moreover, the change in compression savings (measured as BPP) against image quality (measured as PSNR and SSIM) is plotted in Figure 10. As BPP increases, the proposed strategy generates higher-quality compressed images than JPEG, Ref. [18], and Ref. [31].

### 4.3. Computational Complexity Analysis

A promising algorithm obeys low time complexity; we combined JPEG compression with encryption to balance data security and compression efficiency in this study. The time complexity of this algorithm mainly includes three parts: compression, key generation, and encryption. Four steps occur in the JPEG compression algorithm that has been presented thoroughly in Section 2.1, and the computation complexity of JPEG compression is approximately O(MN), where *M* and *N* represent the image’s size. The pseudo-random sequences are generated by the Lorenz hyperchaotic system, in which the time complexity is O(n), wherein n expresses the iterated times of the chaotic system. The encryption algorithm mainly contains five stages: coefficient distribution, block permutation, DC coefficient encryption, non-zero AC coefficient sign transformation, and RSV pair separate–permute–restore–permute, in which the time complexity is O($MN+\frac{MN}{64}+m+k+\frac{r^{2}s}{2}$), where m is the number of DC coefficients, k signifies the number of AC coefficients, r stands for the number of non-zero 8 × 8 blocks, and s indicates the number of RSV pairs in every 8 × 8 block. Therefore, the computational complexity of this algorithm is O($MN+n+MN+\frac{MN}{64}+m+k+\frac{r^{2}s}{2}$), which after simplifying is approximately O($2MN+n+r^{2}s$).

### 4.4. The Effect of Encryption Strategy on Compression

This section presents a detailed analysis of the effect of different encryption methods on compression performance, including file size and execution time. The compression performance should be fully accounted for in the stage of designing the encryption algorithm.

#### 4.4.1. The Effect of DCT Permutation on Compression

**Figure 11:**
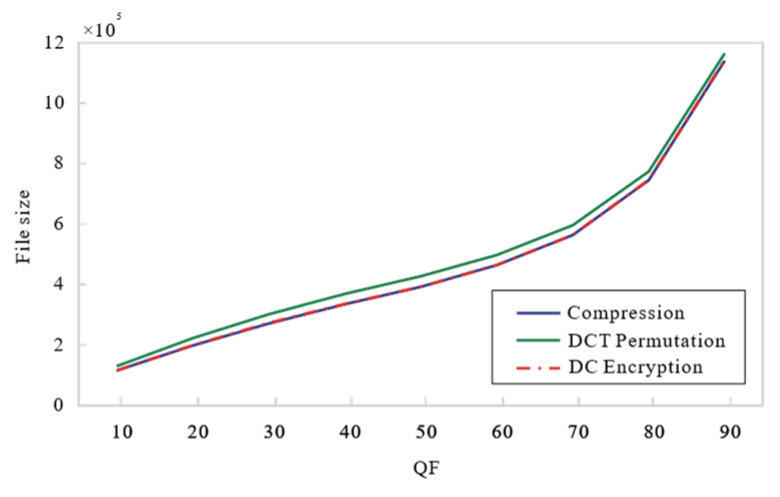
The variation of file size.

**Figure 12:**
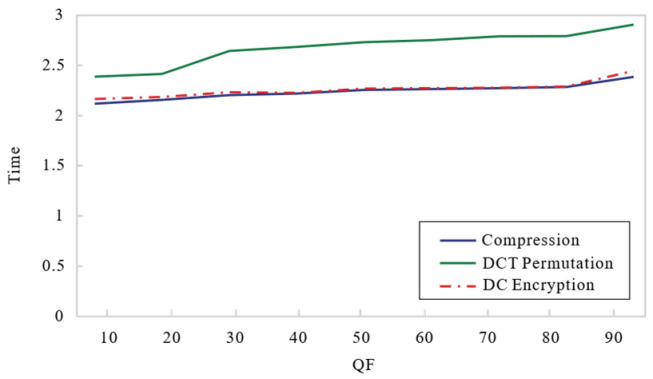
The variation of computational time.

A coefficient permutation strategy based on the generated key is proposed in the DCT transformation phase. A comprehensive analysis is presented in this subsection on the effect of this encryption method on compression, using the common image (Mandrill) as an example to test the variation of bit increase and time overhead with different QF values, which are plotted in Figure 11 and Figure 12. Obviously, the file size and computational overhead are enhanced as QF increases. Among them, the blue solid is the compression baseline, and the green solid expresses the result of coefficient permutation. Experimental results have demonstrated that the proposed scheme incurs a slight increase in bit size and computational time.

#### 4.4.2. The Effect of Shuffling on the DPCM

The block permutation and DC encryption are performed in this study, which may affect the next step. In this subsection, we prove the algorithm’s feasibility using the Mandrill image. As depicted in Figure 11 and Figure 12, the red solid quantizes this encryption result, and all values are basically similar to the compression result that represents the blue solid. The results indicate that the encryption algorithm demonstrates efficacy despite its negligible impact on deposit space and communication costs.

## 5. Conclusions

In this study, we propose a novel scheme that combines compression and encryption. We begin by designing a block splitting method that divides each 16 × 16 block into four 8 × 8 blocks, based on the 16 × 16 Discrete Cosine Transform (DCT), to ensure compatibility with the standard quantization table of the JPEG compression standard. Next, block permutation is applied to disrupt the correlation between blocks. The DC coefficient and nonzero AC coefficients are encrypted using pseudo-random key sequences generated by the Lorenz hyperchaotic system. Additionally, we introduce RSV pair recombination during entropy coding to further break the correlation between adjacent pixels, thereby enhancing data security. All RSV pairs are split into two parts within the blocks. Each block in the first part is paired with a corresponding block in the second part, and the RSV pairs between these two blocks are permuted and recombined. Moreover, an extra RSV pair, representing the displacement of the segmentation point, is embedded within each block of the first part. Compared to existing schemes, only half of the additional RSV pairs are embedded while enhancing the data security and running efficiency. Extensive experiments and various evaluation metrics show that our approach significantly improves compression efficiency and noticeably ruins the pixel correlation to immunize against the various attacks. However, the vulnerability of our algorithm did not fully disrupt intra-block pixel correlations, resulting in low information entropy in ciphertext images. In future work, we will address this limitation by increasing, reducing, or modifying a part of the pixel to break the intra-block pixel correlations.

## Figures and Tables

**Table 1:** Different syntax elements are encrypted to impact performance metrics.

Syntax Elements	Bit Expansion	Efficiency	Perceptual Security	Block Effect	Edge Information
FPV	0.32	High	Low	No	Clear
RPV	2.43	Low	Low	No	Clear
DCADCT	0.01	High	Low	Yes	Clear
ACADCT	2.61	Low	Low	No	Clear
DCAQ	0.04	High	Mediate	Yes	Vaguely observed
ACAQ	1.28	Low	High	Yes	Vaguely observed
DCADPCM	0.04	High	High	Yes	Invisibility
FPV	0.32	High	Low	No	Clear
RPV	2.43	Low	Low	No	Clear
DCADCT	0.01	High	Low	Yes	Clear

**Table 2:** Correlation coefficient for image encrypted with original key and slightly different key.

Image	Algorithm				
	Key_1_	Key_2_	Key_3_	Key_4_	Key_1_
Mandrill	0.000288	0.000098	−0.002164	−0.000320	0.000288
Airplane	0.008365	0.004452	−0.002935	−0.004463	0.008365
Car	−0.000011	0.001881	0.004511	0.000033	−0.000011
Earth	−0.006809	0.006958	−0.004686	−0.003050	−0.006809
House	−0.006800	0.002395	−0.006090	−0.006233	−0.006800
Jelly	−0.004974	−0.001895	−0.009582	0.013820	−0.004974
Peppers	0.009514	0.008712	0.002132	−0.000099	0.009514
Sailboat	0.003398	−0.000293	−0.002472	0.003368	0.003398
Tree	−0.003853	−0.002358	0.000253	−0.002544	−0.003853

**Table 3:** NPCR and UACI of cipher images with one pixel changing.

Image	Proposed Scheme					
	NPCR%			UACI%		
	R	G	B	R	G	B
Mandrill	99.51	99.52	99.50	24.69	25.39	24.39
Airplane	99.50	99.54	99.51	26.49	26.96	25.99
Car	99.49	99.53	99.50	25.55	26.30	25.37
Earth	99.48	99.54	99.49	26.55	27.28	26.22
House	99 54	99.55	99.52	26.74	27.16	26.06
Jelly	99.49	99.56	99.50	26.50	28.45	27.28
Peppers	99.50	99.55	99.51	25.89	27.15	26.01
Sailboat	99.50	99.54	99.49	25.39	26.20	25.28
Tree	99.49	99.53	99.49	25.14	25.81	25.08
Average value of 20 test images	99.45	99.51	99.48	25.86	26.75	25.71

**Table 4:** NPCR and UACI with different algorithms.

Image	Ref. [18]-1						Ref. [18]-2						Ref. [31]					
	NPCR%			UACI%			NPCR%			UACI%			NPCR%			UACI%		
	R	G	B	R	G	B	R	G	B	R	G	B	R	G	B	R	G	B
Mandrill	99.07	99.16	98.86	22.92	23.79	22.01	99.44	99.43	99.36	23.77	24.60	23.20	99.50	99.49	99.56	24.72	23.26	25.82
Airplane	99.41	99.38	99.42	23.01	22.71	22.95	99.21	99.21	99.21	24.37	24.28	23.46	99.62	99.59	99.65	27.89	29.32	29.21
Car	99.37	99.42	99.36	22.38	23.06	21.65	99.42	99.36	99.39	23.58	24.51	23.16	99.54	99.56	99.55	25.51	27.08	25.73
Earth	99.26	99.34	99.26	20.22	20.81	20.16	99.43	99.44	99.42	22.44	23.17	22.14	99.42	99.54	99.39	20.66	26.34	19.90
House	98.50	98.53	98.49	23.42	23.69	22.81	98.83	98.85	98.80	24.27	24.14	23.61	99.44	99.56	99.46	20.26	25.45	25.84
Jelly	98.51	98.51	98.40	24.27	23.36	22.24	98.81	98.91	98.86	25.51	25.56	24.40	99.61	99.61	99.51	26.41	29.33	22.43
Peppers	99.32	99.33	99.33	21.93	22.75	21.03	99.43	99.45	99.43	23.34	24.18	22.44	99.50	99.60	99.66	24.40	31.38	31.83
Sailboat	99.42	99.39	99.34	20.14	20.93	19.44	99.46	99.45	99.46	22.25	23.13	21.83	99.40	99.59	99.63	21.07	30.24	30.07
Tree	98.59	98.63	98.56	22.24	22.07	21.92	99.07	99.17	99.10	23.20	23.74	22.77	99.51	99.64	99.49	24.77	30.60	26.55
Average value of 20 test images	99.15	99.02	99.05	22.23	22.53	21.52	99.28	99.15	99.28	23.64	24.24	23.02	99.53	99.55	99.58	23.93	28.21	26.47

**Table 5:** Correlation coefficients of adjacent pixels.

Image	JPEG			Proposed Scheme		
	H	V	D	H	V	D
Mandrill	0.8602	0.7651	0.7097	0.0797	0.0376	0.0165
Airplane	0.9710	0.9681	0.9437	0.4053	0.1568	0.0991
Car	0.9454	0.9592	0.9129	0.2871	0.0814	0.0766
Earth	0.9769	0.9815	0.9584	0.3573	0.1488	0.1190
House	0.9771	0.9621	0.9419	0.4581	0.1199	0.1013
Jelly	0.9791	0.9821	0.9606	0.5348	0.1846	0.1304
Peppers	0.9202	0.9345	0.9591	0.4539	0.1187	0.0814
Sailboat	0.9779	0.9766	0.0212	0.2466	0.0981	0.0741
Tree	0.9693	0.9517	0.9329	0.1334	0.0235	0.0337
Average value of 20 test images	0.9427	0.9353	0.8229	0.3181	0.1123	0.0765

**Table 6:** Correlation coefficients with different algorithms.

Image	Algorithm			Ref. [18]-1			Ref. [18]-2			Ref. [31]		
	H	V	D	H	V	D	H	V	D	H	V	D
Mandrill	0.0797	0.0376	0.0165	0.5407	0.5437	0.4608	0.1900	0.1708	0.1648	0.0917	0.1560	0.0703
Airplane	0.4053	0.1568	0.0991	0.6720	0.7573	0.5979	0.5289	0.4746	0.4096	−0.0273	0.1242	0.0362
Car	0.2871	0.0814	0.0766	0.5558	0.6928	0.5093	0.4088	0.3776	0.3227	0.1056	0.1487	0.0405
Earth	0.3573	0.1488	0.1190	0.6810	0.7751	0.6174	0.5522	0.4747	0.4018	0.1759	0.1698	0.0665
House	0.4581	0.1199	0.1013	0.6586	0.7591	0.5828	0.4914	0.4895	0.4084	0.1467	0.2014	0.0621
Jelly	0.5348	0.1846	0.1304	0.7967	0.8559	0.7436	0.7380	0.6777	0.5962	0.0644	0.1693	0.0009
Peppers	0.4539	0.1187	0.0814	0.6183	0.7156	0.5399	0.4909	0.4462	0.3568	0.1084	0.1978	0.0019
Sailboat	0.2466	0.0981	0.0741	0.5758	0.6728	0.4959	0.3638	0.3211	0.2474	0.0451	0.0507	0.0021
Tree	0.1334	0.0235	0.0337	0.3761	0.5212	0.3050	0.1963	0.1645	0.1073	−0.0056	0.0367	0.0186
Average value of 20 test images	0.3181	0.1123	0.0765	0.5925	0.6873	0.5239	0.4384	0.3875	0.3024	0.0678	0.1377	0.0351

**Table 7:** The correlation of the plain ‘Mandrill’ image with different algorithms.

File Name	Image	R	G	B
Mandrill	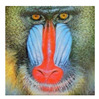	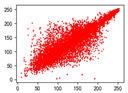	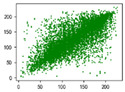	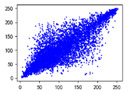
JPEG	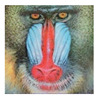	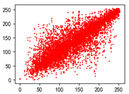	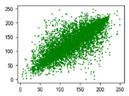	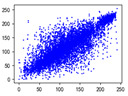
Algorithm	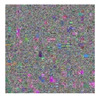	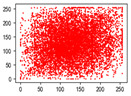	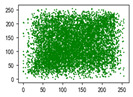	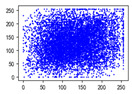
Ref. [18]-1	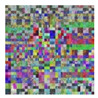	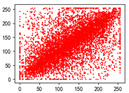	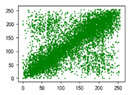	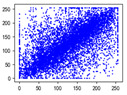
Ref. [18]-2	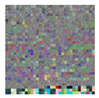	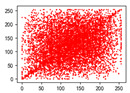	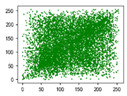	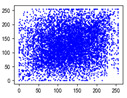
Ref. [31]	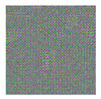	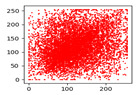	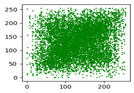	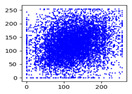

**Table 8:** The results of information entropy with different methods.

Images	Information Entropy											
	R				G				B			
	Algorithm	Ref. [18]-1	Ref. [18]-2	Ref. [31]	Algorithm	Ref. [18]-1	Ref. [18]-2	Ref. [31]	Algorithm	Ref. [18]-1	Ref. [18]-2	Ref. [31]
Mandrill	7.770	7.864	7.832	7.721	7.784	7.905	7.815	7.766	7.753	7.867	7.772	7.65
Airplane	7.809	7.917	7.886	7.712	7.845	7.951	7.904	7.776	7.799	7.903	7.879	7.669
Car	7.777	7.897	7.857	7.717	7.808	7.924	7.857	7.766	7.765	7.879	7.843	7.663
Earth	7.783	7.903	7.871	7.708	7.846	7.936	7.900	7.721	7.774	7.904	7.851	7.633
House	7.812	7.861	7.800	7.717	7.828	7.937	7.866	7.781	7.779	7.892	7.826	7.654
Jelly	7.754	7.843	7.857	7.736	7.894	7.867	7.872	7.727	7.894	7.870	7.850	7.701
Peppers	7.795	7.907	7.847	7.722	7.842	7.934	7.876	7.816	7.792	7.903	7.845	7.688
Sailboat	7.772	7.881	7.832	7.745	7.811	7.928	7.858	7.779	7.756	7.877	7.821	7.719
Tree	7.773	7.876	7.844	7.743	7.796	7.898	7.852	7.771	7.776	7.879	7.837	7.716
Average value of 20 test images	7.782	7.883	7.847	7.724	7.828	7.920	7.866	7.767	7.787	7.886	7.836	7.677

## Data Availability

The data presented in this study are available on request from the corresponding author.
